# Comparing Surface and Fine-Wire Electromyography Activity of Lower Leg Muscles at Different Walking Speeds

**DOI:** 10.3389/fphys.2019.01283

**Published:** 2019-10-10

**Authors:** Annamária Péter, Eva Andersson, András Hegyi, Taija Finni, Olga Tarassova, Neil Cronin, Helen Grundström, Anton Arndt

**Affiliations:** ^1^Neuromuscular Research Center, Faculty of Sport and Health Sciences, University of Jyväskylä, Jyväskylä, Finland; ^2^The Swedish School of Sport and Health Sciences (GIH), Stockholm, Sweden; ^3^Department of Neuroscience, Karolinska Institute, Stockholm, Sweden; ^4^Department of Radiology, Capio S:t Göran’s Hospital, Stockholm, Sweden; ^5^Department of Clinical Science, Intervention and Technology, Karolinska Institute, Stockholm, Sweden

**Keywords:** bipedal locomotion, ankle plantar flexor muscles, surface electromyography, EMG, intramuscular electromyography

## Abstract

Ankle plantar flexor muscles are active in the stance phase of walking to propel the body forward. Increasing walking speed requires increased plantar flexor excitation, frequently assessed using surface electromyography (EMG). Despite its popularity, validity of surface EMG applied on shank muscles is mostly unclear. Thus, we examined the agreement between surface and intramuscular EMG at a range of walking speeds. Ten participants walked overground at slow, preferred, fast, and maximum walking speeds (1.01 ± 0.13, 1.43 ± 0.19, 1.84 ± 0.23, and 2.20 ± 0.38 m s^–1^, respectively) while surface and fine-wire EMG activities of flexor hallucis longus (FHL), soleus (SOL), medial gastrocnemius (MG) and lateral gastrocnemius (LG), and tibialis anterior (TA) muscles were recorded. Surface and intramuscular peak-normalised EMG amplitudes were compared for each muscle and speed across the stance phase using Statistical Parametric Mapping. In FHL, we found differences around peak activity at all speeds except fast. There was no difference in MG at any speed or in LG at slow and preferred speeds. For SOL and LG, differences were seen in the push-off phase at fast and maximum walking speeds. In SOL and TA, surface EMG registered activity during phases in which intramuscular EMG indicated inactivity. Our results suggest that surface EMG is generally a suitable method to measure MG and LG EMG activity across several walking speeds. Minimising cross-talk in FHL remains challenging. Furthermore, SOL and TA muscle onset/offset defined by surface EMG should be interpreted cautiously. These findings should be considered when recording and interpreting surface EMG of shank muscles in walking.

## Introduction

Electromyography is often used in research and clinical environments to examine muscle excitations in normal and pathological conditions. There are two predominant forms of EMG measurement; surface and intramuscular EMG (Farina and Negro, 2012). Non-invasive surface EMG is widely used for superficial, large, and easily accessible muscles. With surface EMG, excitation level is acquired from a large area including several motor unit populations (Roy et al., 1986). Despite its popularity and relatively simple use, surface EMG has some inherent limitations. For example, selective recordings from deep muscles are not possible. Furthermore, due to the relatively large pick-up area of the electrodes, unwanted signals can be recorded from adjacent or deep muscles (Perry et al., 1981; Dimitrova et al., 2002; Lowery et al., 2003), an error source termed cross-talk. Cross-talk needs to be rigorously considered since it may lead to the misinterpretation of the recorded EMG signal (De Luca, 1997). General suggestions for minimising cross-talk include proper surface electrode location (Roy et al., 1986), and proper electrode size and inter-electrode distance (Merletti et al., 2001). Proper surface electrode location is also important since electrodes located close to the innervation zone or the tendon region may cause large signal amplitude variation (Sadoyama et al., 1985; Roy et al., 1986; Rainoldi et al., 2000, 2004; Farina et al., 2001; Merletti et al., 2001). Furthermore, during muscle contractions the muscle moves under the skin and the electrodes, which may also have considerable effects on the recorded surface EMG signal (De Luca, 1997; Rainoldi et al., 2000; Farina et al., 2001).

Intramuscular EMG is an invasive method and is therefore seldom used. It is primarily used to study deep muscles (Andersson et al., 1997; Õunpuu et al., 1997; Onishi et al., 2000), and muscles that have a small cross-sectional area (Andersson et al., 1997; Sutherland, 2001). Furthermore, special skills are required to insert the electrodes, and this process takes longer compared to the placement of surface electrodes (Õunpuu et al., 1997). A major advantage of intramuscular EMG compared to surface EMG is that it is suitable to selectively detect EMG signals of a muscle during static and dynamic conditions, while minimising cross-talk (Perry et al., 1981; De Luca and Merletti, 1988; De Luca, 1997; Onishi et al., 2000). By inserting fine wires intramuscularly, the EMG recording wires will follow the movement of the muscle underneath the skin during dynamic contractions (e.g., in walking) (Hodges and Gandevia, 2000; Chapman et al., 2010). Since it does not seem to affect human gait patterns (Winchester et al., 1996), this method can be used to detect EMG activity in human walking.

Human walking is a common movement requiring substantial work from lower leg muscles, especially in the stance phase. Therefore, recording EMG activity from different muscles of the lower leg with high accuracy is of primary interest in gait studies. Since both surface and intramuscular EMG methods aim to examine muscle excitation timing and amplitude, previous studies have used intramuscular EMG to validate the idea that surface EMG signals contain no or negligible cross-talk (i.e., measure from the target muscle explicitly) (Kadaba et al., 1985; Bogey et al., 2000, 2003). These studies focused on the SOL and TA muscles only, explicitly measured during gait at self-selected speed. However, the speed of locomotion seems to affect muscle function and kinematics (Lelas et al., 2003; Cronin et al., 2013). It is assumed that increased walking speed requires increased ankle plantar flexor work (Neptune et al., 2008). The ankle plantar flexors’ relative contribution to propulsion also changes with speed besides a general increase in surface EMG activity in these muscles (Cronin et al., 2013). To the best of our knowledge, there is no study yet in which the validity of the surface EMG method was examined for lower leg muscles apart from SOL and TA across a range of walking speeds.

Thus, in this study we simultaneously recorded surface and intramuscular EMG activity from flexor hallucis longus (FHL), SOL, MG, LG, and TA muscles at different walking speeds to examine whether EMG signals recorded with the two methods show differences in EMG amplitude at any point across the stance phase of walking. We hypothesised that there would be no difference between surface and intramuscular EMG activity of FHL, SOL, MG, LG, and TA muscles at relatively slow walking speeds, but disagreement between these methods would appear at faster speeds.

## Materials and Methods

### Participants

After convenience-based sampling, 10 healthy, physically active individuals (six males, four females; age 29.6 ± 7.4 years, height 174.0 ± 12.5 cm, body mass 70.6 ± 12.7 kg, body mass index: 23.14 ± 1.7 m^–2^ kg) without history of neuromuscular disorders or previous/current leg injuries gave written consent to participate in this study. The study was approved by the Stockholm regional ethics committee (Approval No.: 2017/261-31/4) and was performed in agreement with the Declaration of Helsinki.

### Study Protocol

**FIGURE 1:**
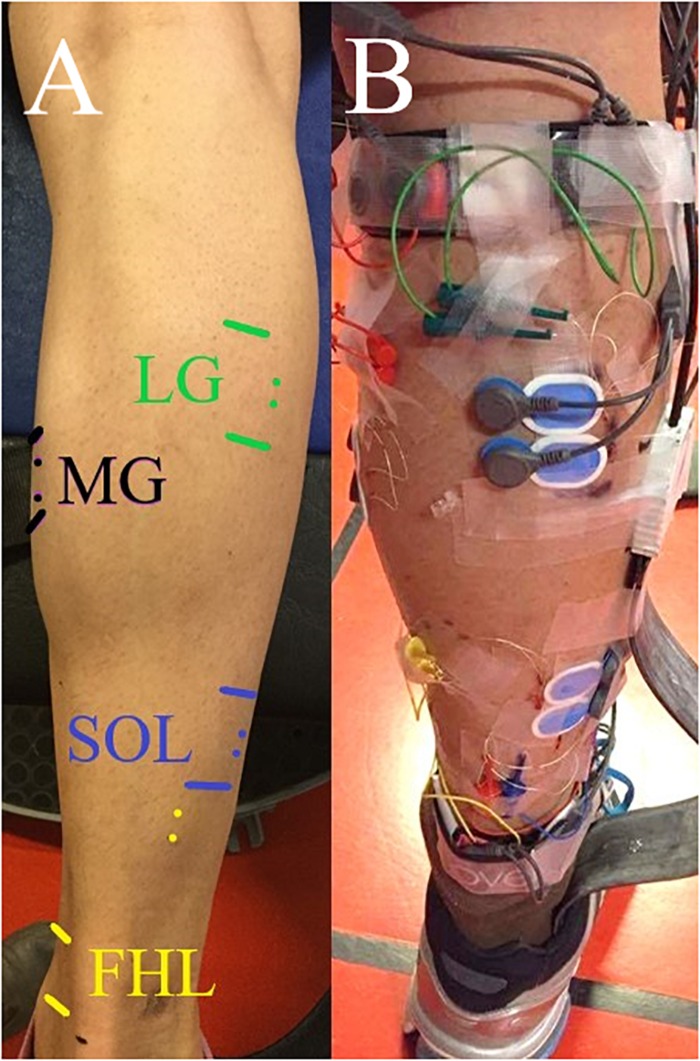
**(A)** The locations of the surface and intramuscular electrodes on the investigated leg’s flexor hallucis longus (FHL), soleus (SOL), and medial and lateral gastrocnemii (MG and LG). Surface electrodes were placed between the horizontal lines; intramuscular electrodes were inserted approximately where the dots are shown. **(B)** The investigated leg with the surface and intramuscular electrodes attached. Tibialis anterior (TA) was also measured (not shown).

Participants first attended a familiarisation session where they were acquainted with the study protocol and surface EMG electrode locations were marked with permanent pen (Figure 1A). The main testing session was 1–3 days later, where after standardised warm-up in a dynamometer (submaximal and maximal isometric plantar flexion and dorsiflexion contractions) and 5 min preferred-speed walking, data collection started. First, participants walked at their self-selected steady speed along the measurement area five times in typical cushioned running shoes while surface and intramuscular EMG activities from the right shank and ground reaction forces were recorded. Out of the five trials, the slowest and fastest trials were excluded and the speeds of the remaining three trials were averaged to define preferred walking speed of each individual. Then they walked at three randomly ordered speeds, which were 30% slower and 30% faster than preferred walking speed (within ±5% of target speed), and maximum walking speed where participants were asked to walk as fast as they could. Three successful trials were recorded at each speed. Overground walking trials started and terminated 2–3 m before and after the measurement area, respectively, to aim for a steady speed over the 7 m measurement area. To define walking speeds, custom-made photocells were installed at the beginning and end of the measurement area. Participants reported no discomfort and their walking pattern seemed normal during the recordings.

### Instrumentation

#### Surface and Intramuscular EMG Activity

After shaving, lightly abrading, and cleaning the skin with alcohol, silver–silver chloride Ambu BlueSensor N (Ambu A/S, Ballerup, Denmark) surface electrodes were put on the examined muscles in bipolar configuration (Figure 1B). FHL electrodes were placed behind the medial malleolus where the muscle belly was superficial as defined by ultrasonography (Echo Blaster 128; Telemed, Vilnius, Lithuania) [detailed description in Péter et al. (2015)]. For MG, LG, and TA muscles, the SENIAM recommendations were followed with slight adjustments if individual-specific muscle morphologies required. According to SENIAM (Hermens et al., 2000), SOL surface electrodes are recommended to be placed on the medial side of the shank at two-third of the line between medial femur condyle to medial malleolus. However, it has been shown previously that surface electrodes placed as recommended were prone to register activity from muscles other than SOL during self-selected speed walking (Bogey et al., 2000). Therefore, in this study we placed SOL surface electrodes laterally, at the same proximal–distal location. For FHL, inter-electrode distance was decreased to 16 mm to minimise cross-talk (Bojsen-Moller et al., 2010; Masood et al., 2014), while inter-electrode distance for other muscles was 22 mm. Furthermore, we also measured the distance between SOL insertion and distal FHL muscle tendon junction to examine if the magnitude of the space where surface electrodes can be placed has any effects on the surface EMG activity compared to intramuscular EMG.

Intramuscular electrodes were inserted by an experienced radiologist (co-author HG) under real-time, high-resolution B-mode, and Doppler ultrasonography (Logiq E9, GE, United States) guidance. After cleaning the skin with alcohol, Teflon-coated seven-stranded silver hook-wire electrodes were inserted in bipolar configuration with an inter-tip distance of ≈5 mm (wire diameter 0.25 mm, stripped length of 2 mm forming the recording surface, previously sterilised in an autoclave), two in each muscle with hypodermic needles (diameter 0.8 mm, after wire insertion the needles were carefully withdrawn). The recording ending of the intramuscular EMG electrodes for SOL, MG, LG, and TA muscles were inserted underneath the surface electrodes. FHL intramuscular electrodes were inserted 5–10 cm proximal to the surface electrodes, on the lateral side of the shank, depending on the thickness of the muscle belly and vascularisation (Figure 1A).

Surface and intramuscular EMG activity of all muscles was simultaneously recorded using a telemetric system (MyoSystem 1400A, Noraxon Inc., Scottsdale, AZ, United States) with a sampling frequency of 3000 Hz. Signals were transmitted wirelessly to an A/D converter (Cambridge Electronic Design, Cambridge, United Kingdom) that was connected to a personal computer. Digital signals were collected and visualised online in Spike2 software (Cambridge Electronic Design, Cambridge, United Kingdom). A single surface reference electrode (silver–silver chloride Ambu BlueSensor N, Ambu A/S, Ballerup, Denmark) was attached to the skin over the medial aspect of the tibia bone.

#### Gait Events

A plantar pressure insole was placed in the right shoe to define the timing of HC and TO for all steps (Pedar-X 99-sensor in-shoe dynamic pressure measuring system, Novel Inc., Munich, Germany, 100 Hz sampling frequency). Data were sent to a personal computer via Bluetooth to record in the Pedar software. To ensure that insoles did not move in the shoes between trials, the big toe was pressed down by an assistant before each trial and we ensured that the same pressure sensors were activated this way (Péter et al., 2015).

Halfway along the walking distance of the measurement area, two 0.6 m × 0.4 m 3-D force platforms (Kistler type 9281EA, Kistler AG, Winterthur, Switzerland) were installed in series. Three-dimensional ground reaction force data were collected from the right leg, one stride per trial to define the start of the push-off phase. Walking trials were repeated if the participant’s right leg did not hit at least one of the force platforms. Data were recorded with Qualisys Track Manager software (Qualisys AB, Sweden), with a sampling frequency of 3000 Hz.

To synchronise data acquisition a trigger signal was sent to the Spike software from the Pedar system at the start of each recording. This trigger signal was sent simultaneously to the Qualisys Track Manager software, which started recordings in this software.

### Analysis

#### Gait Events

First, HC and TO timings were defined for each step over the measurement area in Matlab (MathWorks Inc., Natick, MA, United States) as follows. Vertical force as the sum of forces measured with all sensors of the insole was first extrapolated to 3000 Hz to coordinate with ground reaction force data. Then, HC and TO were defined for the steps on the force plates based on a 10 N vertical ground reaction force threshold (Osis et al., 2016). The timings based on ground reaction force helped to define thresholds for vertical forces measured with the insole to minimise errors due to potential mismatch between the size of the foot and the insole. Based on this subject- and task-specific threshold, HC and TO were defined for all steps from steady-speed trials within the measurement area (i.e., steps where force signals were recorded by pressure insoles only, as well as those recorded by the force plates). EMG activity was analysed between HC and TO for all steps as detailed below.

**FIGURE 2:**
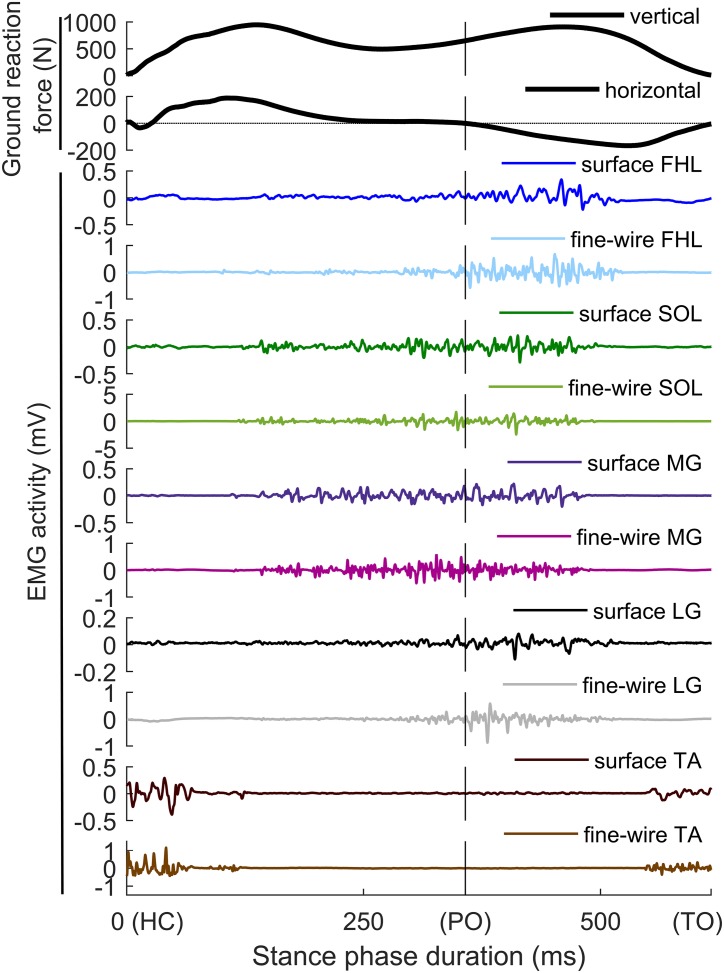
Raw data from one step of one participant recorded during preferred speed walking. The top two panels show ground reaction forces (vertical and horizontal) measured with the force platform. The remaining panels show surface and intramuscular EMG activity of flexor hallucis longus (FHL), soleus (SOL), medial gastrocnemius (MG), lateral gastrocnemius (LG), and tibialis anterior (TA). HC, heel contact; PO, push-off start; TO, toe off.

**FIGURE 3:**
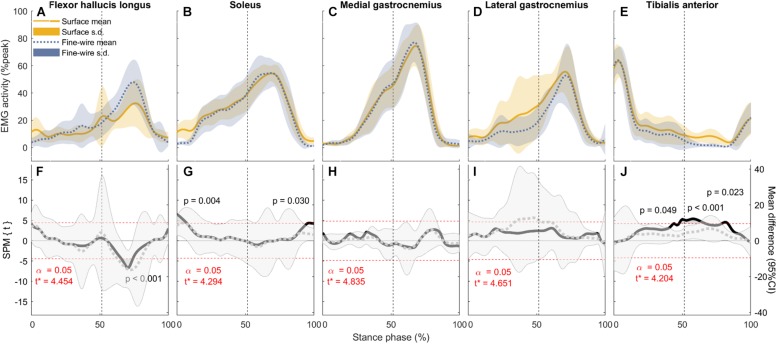
Electromyography (EMG) activity (mean and standard deviation) of each muscle (**A–E**: normalised to peak activity in preferred speed walking, %peak) and the corresponding comparisons between surface and intramuscular EMG recordings **(F–J)** in the stance phase of slow walking (1.01 ± 0.13 m s^–1^). Vertical dotted lines represent the beginning of push-off. Panels **(F–J)**: SPM{*t*} statistics for two-tailed *t*-tests (solid black lines, left *y*-axis), and mean difference with 95% confidence intervals (grey dotted lines with shaded areas). Dashed red lines are critical thresholds (*t*^∗^) calculated for α significance level defining supra-threshold clusters for SPM{*t*} trajectories. *P-*values were calculated for each supra-threshold cluster.

**FIGURE 4:**
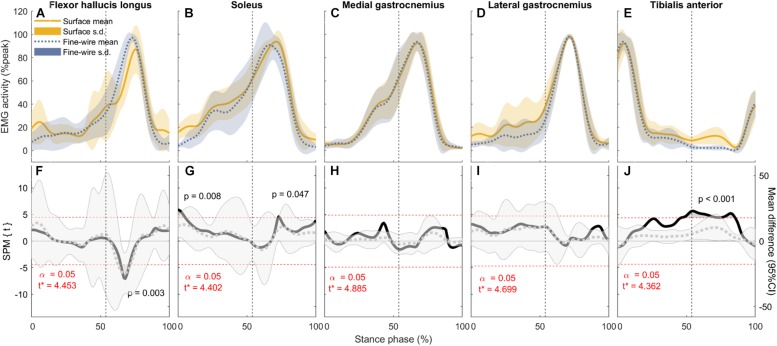
Electromyography (EMG) activity (mean and standard deviation) of each muscle (**A–E**, normalised to peak activity in preferred speed walking, %peak) and the corresponding comparisons between surface and intramuscular EMG recordings **(F–J)** in the stance phase of preferred speed walking (1.43 ± 0.19 m s^–1^). Vertical dotted lines represent the beginning of push-off. Panels **(F–J)**: SPM{*t*} statistics for two-tailed *t*-tests (solid black lines, left *y*-axis), and mean difference with 95% confidence intervals (grey dotted lines with shaded areas). Dashed red lines are critical thresholds (*t*^∗^) calculated for α significance level defining supra-threshold clusters for SPM{*t*} trajectories. *P*-values were calculated for each supra-threshold cluster.

**FIGURE 5:**
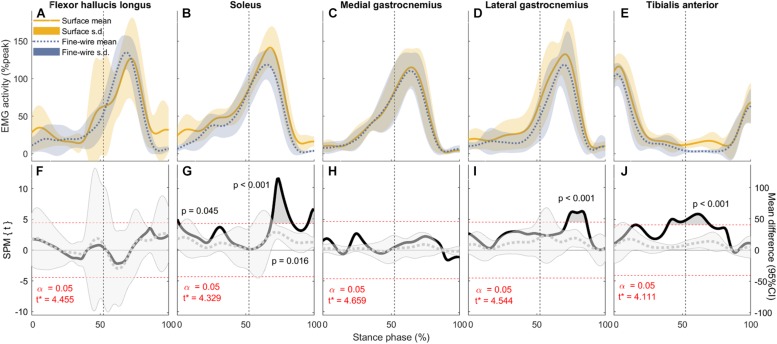
Electromyography (EMG) activity (mean and standard deviation) of each muscle (**A–E**: normalised to peak activity in preferred speed walking, %peak) and the corresponding comparisons between surface and intramuscular EMG recordings **(F–J)** in the stance phase of fast walking (1.84 ± 0.23 m s^–1^). Vertical dotted lines represent the beginning of push-off. Panels **(F–J)** SPM{*t*} statistics for two-tailed *t*-tests (solid black lines, left *y*-axis), and mean difference with 95% confidence intervals (grey dotted lines with shaded areas). Dashed red lines are critical thresholds (*t*^∗^) calculated for α significance level defining supra-threshold clusters for SPM{*t*} trajectories. *P*-values were calculated for each supra-threshold cluster.

**FIGURE 6:**
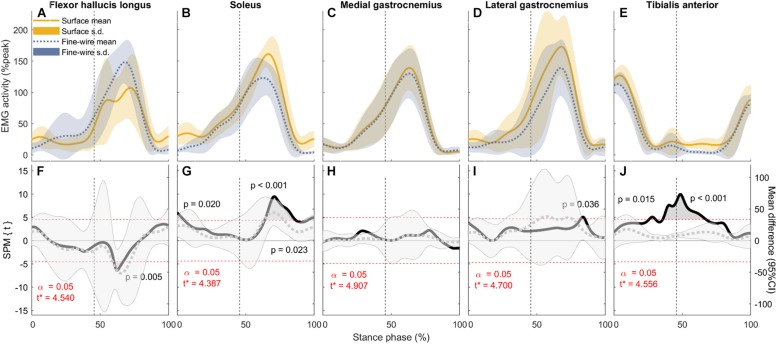
Electromyography (EMG) activity (mean and standard deviation) of each muscle (**A–E**: normalised to peak activity in preferred speed walking, %peak) and the corresponding comparisons between surface and intramuscular EMG recordings **(F–J)** in the stance phase of maximum speed walking (2.20 ± 0.38 m s^–1^). Vertical dotted lines represent the beginning of push-off. Panels **(F–J)** SPM{*t*} statistics for two-tailed *t*-tests (solid black lines, left *y*-axis), and mean difference with 95% confidence intervals (grey dotted lines with shaded areas). Dashed red lines are critical thresholds (*t*^∗^) calculated for α significance level defining supra-threshold clusters for SPM{*t*} trajectories. *P*-values were calculated for each supra-threshold cluster.

For the steps that occurred on force plates, push-off initiation was defined as the time when the anterior–posterior ground reaction force crossed 0 N and became an anterior, propulsive force, for visualisation (Figures 2–6). Sub-phases of the stance were defined as early stance (0–16.5%), mid stance (16.5–50%), late stance (50–83%), and pre-swing (83–100%) based on previous literature (Neptune et al., 2001), to assist data interpretation.

#### EMG Activity

Medial gastrocnemius surface EMG from one and LG surface EMG from another participant were not recorded due to technical issues. Additionally, one of the participants did not perform maximum walking speed trials. All other recordings were included in the analysis.

Electromyography signals of all muscles were analysed in the stance phase of the step cycles as defined above. EMG analyses were conducted in Matlab. Surface and intramuscular EMG signals were band-pass filtered between 20 and 500 Hz using a zero-lag 4th order Butterworth filter. The filtered and rectified signals were smoothed with a 10 Hz zero-lag low-pass filter (4th order Butterworth). Signals for each stance phase were time-normalised (1–101 frames) using linear interpolation. Subsequently, EMG signals were averaged within every walking condition for each participant and muscle. To decrease inter-individual variability, EMG signals were normalised to the peak activity of preferred speed walking (Cronin et al., 2015). These time- and peak-normalised signals were included in further analysis as detailed below.

### Statistical Analysis

We used SPM (Friston, 2007) to statistically examine the difference between surface and intramuscular EMG amplitudes at each point of the time-normalised stance phase. SPM analysis was performed in Matlab using the open-source spm1d code (v.M0.1^1^). SPM two-tailed paired *t*-tests were used to compare surface and intramuscular EMG signal curves of all muscles during slow, preferred, fast, and maximum walking speeds as follows. Firstly, the scalar output statistic SPM{*t*} was calculated forming a Statistical Parametric Map. SPM{*t*} is a scalar trajectory variable, that shows the magnitude of differences between surface and intramuscular EMG signals. The magnitudes of the differences were also expressed as mean difference ± 95% CI. In order to test the null hypothesis, we calculated the critical threshold at which only α% (set to 5%) of smooth random curves would be expected to traverse. This critical threshold calculation is based on estimates of trajectory smoothness via temporal gradients (Friston, 2007) and, based on that smoothness, Random Field Theory expectations regarding the field-wide maximum (Adler and Taylor, 2007). EMG time-series were considered significantly different if any values of SPM{*t*}exceeded the critical threshold. In the final step, cluster specific *p*-values were calculated.

## Results

Walking speeds were 1.01 ± 0.13, 1.43 ± 0.19, 1.84 ± 0.23, and 2.20 ± 0.38 m s^–1^ (mean ± standard deviation), and number of steps included in the analysis were 14 ± 3, 12 ± 3, 11 ± 2, and 9 ± 1 (median ± interquartile range) at slow, preferred, fast, and maximum walking speeds, respectively. Stance phase durations were 0.81 ± 0.06, 0.67 ± 0.06, 0.58 ± 0.04, and 0.5 ± 0.04 s (mean ± standard deviation) at slow, preferred, fast, and maximum walking speeds, respectively. The space between SOL insertion and distal FHL muscle–tendon junction where surface electrodes could be placed was 3.22 cm on average, ranging from 2.5 to 4.7 cm.

Figure 2 shows typical EMG signals from the five muscles recorded both with surface and intramuscular electrodes. Mean EMG activity results for all muscles and walking speeds are presented for the whole group of participants in Figures 3–6 and for each individual in Figures 7–11.

**FIGURE 7:**
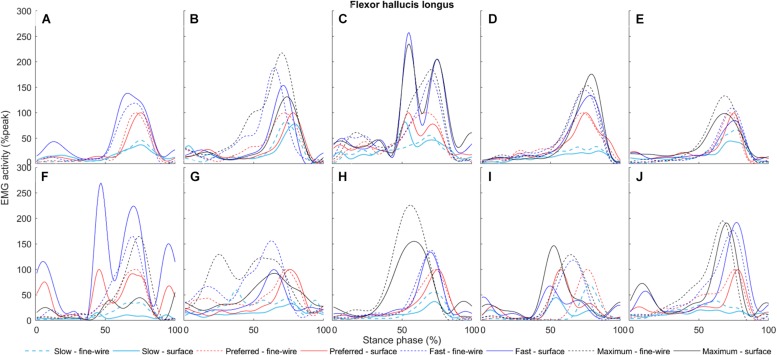
Flexor hallucis longus surface (solid) and intramuscular (dashed) electromyography (EMG) activity at slow, preferred, fast, and maximum walking speeds (1.01 ± 0.13, 1.43 ± 0.19, 1.84 ± 0.23, and 2.20 ± 0.38 m s^–1^, respectively) in the stance phase for each individual (**A–J**, respectively). The amplitudes of smoothed and time-normalised EMG curves were normalised to peak activity in preferred speed walking (%peak). Each curve represents the average of all steps (number of steps included in the analysis were 14 ± 3, 12 ± 3, 11 ± 2, and 9 ± 1, median ± interquartile range, at slow, preferred, fast, and maximum walking speeds, respectively) at a given walking speed.

**FIGURE 8:**
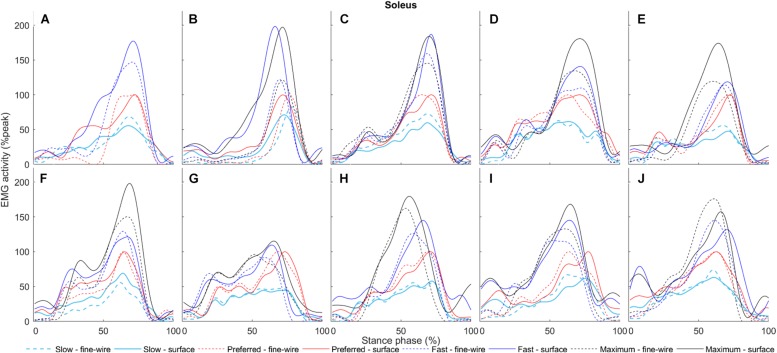
Soleus surface (solid) and intramuscular (dashed) electromyography (EMG) activity at slow, preferred, fast, and maximum walking speeds (1.01 ± 0.13, 1.43 ± 0.19, 1.84 ± 0.23, and 2.20 ± 0.38 m s^–1^, respectively) in the stance phase for each individual (**A–J**, respectively). The amplitudes of smoothed and time-normalised EMG curves were normalised to peak activity in preferred speed walking (%peak). Each curve represents the average of all steps at a given walking speed.

**FIGURE 9:**
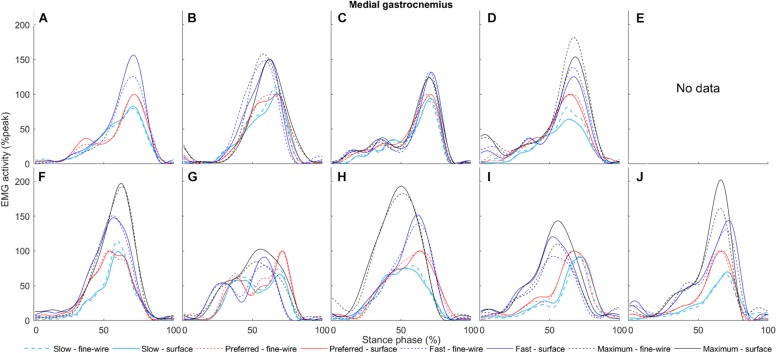
Medial gastrocnemius surface (solid) and intramuscular (dashed) electromyography (EMG) activity at slow, preferred, fast, and maximum walking speeds (1.01 ± 0.13, 1.43 ± 0.19, 1.84 ± 0.23, and 2.20 ± 0.38 m s^–1^, respectively) in the stance phase for each individual (**A–J**, respectively). The amplitudes of smoothed and time-normalised EMG curves were normalised to peak activity in preferred speed walking (%peak). Each curve represents the average of all steps (number of steps included in the analysis were 14 ± 3, 12 ± 3, 11 ± 2, and 9 ± 1, median ± interquartile range, at slow, preferred, fast, and maximum walking speeds, respectively) at a given walking speed.

**FIGURE 10:**
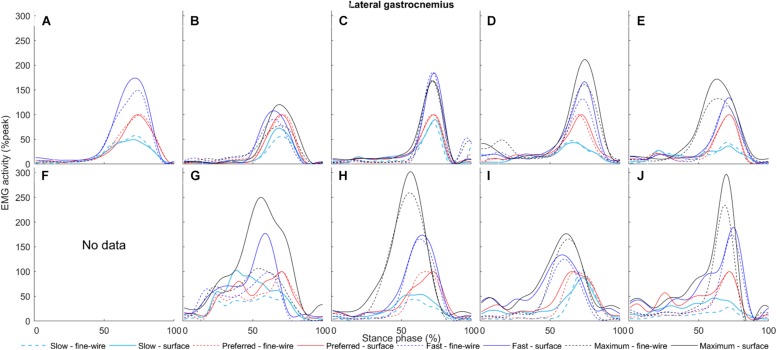
Lateral gastrocnemius (solid) and intramuscular (dashed) electromyography (EMG) activity at slow, preferred, fast, and maximum walking speeds (1.01 ± 0.13, 1.43 ± 0.19, 1.84 ± 0.23, and 2.20 ± 0.38 m s^–1^, respectively) in the stance phase for each individual (**A–J**, respectively). The amplitudes of smoothed and time-normalised EMG curves were normalised to peak activity in preferred speed walking (%peak). Each curve represents the average of all steps (number of steps included in the analysis were 14 ± 3, 12 ± 3, 11 ± 2, and 9 ± 1, median ± interquartile range, at slow, preferred, fast, and maximum walking speeds, respectively) at a given walking speed.

**FIGURE 11:**
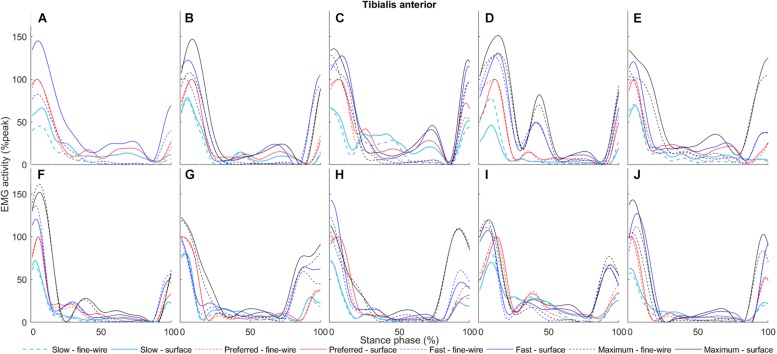
Tibialis anterior surface (solid) and intramuscular (dashed) electromyography (EMG) activity at slow, preferred, fast, and maximum walking speeds (1.01 ± 0.13, 1.43 ± 0.19, 1.84 ± 0.23, and 2.20 ± 0.38 m s^–1^, respectively) in the stance phase for each individual (**A–J**, respectively). The amplitudes of smoothed and time-normalised EMG curves were normalised to peak activity in preferred speed walking (%peak). Each curve represents the average of all steps (number of steps included in the analysis were 14 ± 3, 12 ± 3, 11 ± 2, and 9 ± 1, median ± interquartile range, at slow, preferred, fast, and maximum walking speeds, respectively) at a given walking speed.

In FHL we found differences in late stance at slow, preferred, and maximum speed walking (slow: 65.7–74% of stance phase, *p* < 0.001, preferred: 64.8–70.9%, *p* = 0.003, maximum speed walking: 60.1–66.7%, *p* = 0.005). There was no difference in FHL in fast walking. However, we found subject-specific differences between surface and intramuscular EMG patterns in FHL at all walking speeds (Figure 7).

In SOL, there were differences in early stance at all walking speeds (slow: 0–7.3%, *p* = 0.004, preferred: 0–5.3%, *p* = 0.008, fast: 0–1.4%, *p* = 0.045, maximum: 0–5.2%, *p* = 0.02), and in late stance at all speeds except slow (preferred: 72.7–73.6%, *p* = 0.047, fast: 68.5–84.2%, *p* < 0.001, maximum: 63.8–86.6%, *p* < 0.001). There were also a differences in pre-swing at all speeds except preferred speed (slow: 95.3–98.6%, *p* = 0.03, fast: 68.5–84.2% and 95.3–100%, *p* < 0.001 and *p* = 0.016, respectively, maximum: 63.8–86.6% and 95.2–100%, *p* < 0.001 and *p* = 0.023, respectively).

In TA, there were differences in mid stance at all walking speeds (slow: 43.7–44.3 and 47.5–67.2%, *p* = 0.049 and *p* < 0.001, respectively, preferred: 45.9–85.3%, *p* < 0.001, fast: 39.4–70.2%, *p* < 0.001, maximum: 25.7–30.4 and 35.6–67.2%,*p* = 0.015 and *p* < 0.001, respectively). Similarly, there were differences in late stance at all walking speeds (slow: 47.5–67.2 and 78.6–83.3%, *p* < 0.001 and *p* = 0.023, respectively, preferred: 45.9–85.3%, *p* < 0.001, fast: 39.4–70.2%, *p* < 0.001, maximum: 35.6–67.2%, *p* < 0.001). There were also a differences in pre-swing at slow and preferred speed (slow: 78.6–83.3%, *p* = 0.023, preferred: 45.9–85.3%, *p* < 0.001).

In MG there was no difference between surface and intramuscular EMG at any walking speed, or at slow or preferred speed walking in LG. However, at faster speeds we found differences in LG in late stance and pre-swing (fast: 71.8–86.3%, *p* < 0.001, maximum speed walking: 82–85%, *p* = 0.036).

## Discussion

This study compared surface and intramuscular EMG activity of key plantar flexor muscles in walking at different speeds by comparing the amplitudes of surface and intramuscular EMG recordings from healthy participants. In FHL, we found differences in the late stance phase at all speeds except fast, therefore questioning the validity of surface EMG for this muscle. In SOL and TA, surface EMG registered activity during phases in which intramuscular EMG indicated inactivity. For SOL and LG, differences between the two EMG methods were also seen around peak activity at relatively fast walking speeds. These results suggest that surface EMG signals were influenced by cross-talk in these cases. No differences in EMG amplitudes were found between the two methods for MG at any speed and LG at slow and preferred speeds, supporting the validity of surface EMG for these muscles within the range of walking speeds studied.

Early studies indicate that FHL is active in the stance phase of walking (Perry, 1992). This study confirmed that FHL is mainly active in push-off, therefore indicating a potential role of this muscle in propulsion. Bojsen-Moller et al. (2010) demonstrated that surface EMG recordings from FHL are possible with minimal cross-talk in submaximal contractions when electrodes are placed behind the medial malleolus with 16 mm interelectrode distance. To further improve electrode location accuracy, we used ultrasonography guidance (Péter et al., 2015, 2017). Despite careful electrode location and the decreased inter-electrode distance, the current study revealed significant subject-specific differences between surface and intramuscular EMG patterns in FHL at a range of walking speeds. Indeed, Figure 7 shows that surface and intramuscular EMG activity of participants A, D, E, and H follow similar patterns in contrast to the six other participants. The reason why the amplitudes of surface and intramuscular EMG recordings are in agreement for some but not other participants could be manifold. One explanation is that the mechanical behaviour of FHL muscle–tendon complex seems to be individual-specific, and this presumably affects surface EMG recordings due to tissue motion underneath the skin. For example, in cadaver gait simulations, Hofmann et al. (2013) reported large differences between FHL tendon excursion in the stance phase ranging between 4.31 and 10.16 mm (mean = 7.18 mm, *n* = 8). Regarding muscle mechanics *in vivo*, we previously detected high inter-individual differences in FHL fascicle length change at similar walking speeds to those applied in the current study (Péter et al., 2017). It should also be mentioned that the defined space where surface electrodes were placed (distance between SOL insertion and distal FHL muscle tendon junction) was as short as 3.22 cm (ranging from 2.5 to 4.7 cm), increasing the potential for cross-talk, although data from some participants with relatively shorter distance presented good agreement between methods compared to others with more space for electrodes. Other factors such as changes in skin impedance and inter-individual differences in subcutaneous tissue thickness behind the medial malleolus can further influence surface EMG recordings. Some of the potential factors that increase cross-talk are challenging to examine and control, therefore based on our current knowledge we suggest using intramuscular instead of surface EMG to define the EMG activity for FHL muscle.

Soleus is mostly active from mid stance to the beginning of the pre-swing phase during walking, and is inactive in the swing phase (Perry, 1992; Cuccurullo, 2004). Previous studies found no intramuscular EMG activity in the swing phase but some activity was seen from surface EMG in preferred-speed walking, suggesting potential cross-talk from TA (Bogey et al., 2000, 2003). Although surface and intramuscular EMG activity followed similar patterns in the active phase, both studies [see Figure 1 in Bogey et al. (2000) and Figure 2 in Bogey et al. (2003)] showed higher SOL surface activity in early stance and at the end of the pre-swing phase compared to intramuscular EMG at preferred speed walking. Concurrently, we detected activity with surface EMG in early stance at all speeds and in the pre-swing phase at all speeds except preferred speed while there was no activity with intramuscular EMG, suggesting that surface EMG was subject to cross-talk. Furthermore, we found differences between surface and intramuscular EMG signal amplitudes in SOL around peak activity at preferred, fast, and maximum speed walking. Our results show that at all walking speeds when surface EMG electrodes are placed laterally, they are prone to detect activity in those phases in which intramuscular EMG indicates that the muscle is actually inactive. This is similar to previous findings (Bogey et al., 2000, 2003) where medial surface electrode placement was used. Thus, SOL surface EMG activity may be affected by cross-talk and based on these results, SOL onset/offset during walking defined by surface EMG signals should be interpreted cautiously. Based on our results SOL surface electrodes should be placed over the skin with great caution. After checking SOL surface electrode location based on SENIAM recommendations, the location should be reconsidered based on muscle belly thickness to decrease cross-talk and moved to a location where SOL muscle belly is larger but also sufficiently far from MG and LG.

Similar to previous reports (Perry, 1992; Cuccurullo, 2004), the gastrocnemii muscles were activated in the stance phase. In MG we found no difference between surface and intramuscular EMG amplitudes at any walking speeds, suggesting that surface EMG is a suitable method with the used surface electrode location and inter-electrode distance. Compared to other plantar flexors, full agreement between methods across a range of walking speeds can be explained by the large cross-sectional area of MG, which enables electrodes to be placed a sufficient distance from other muscles, thereby minimising cross-talk. Although LG has substantially lower volume (Ward et al., 2009), surface EMG seems valid at slow and preferred speed walking, but small differences were seen in three participants (participant G, I, and J; Figure 10). However, LG surface electrodes seem to pick up some activity from surrounding muscles at faster speeds. This may be due to increased activity of neighbouring muscles and alteration in intermuscular coordination strategies as walking speed increases (Cronin et al., 2013). Our results suggest that surface EMG cross-talk is minimised for LG, at least at slow and preferred speed walking.

Previous studies showed two distinct peaks in intramuscular TA EMG activity in the stance phase of walking, near heel-strike, and TO, respectively, whereas TA was not active in the mid stance (or mid-swing) phases in healthy individuals (Gray and Basmajian, 1968). These findings are all in agreement with our intramuscular results. However, we detected surface but not intramuscular EMG activity in the mid and late stance phases at all walking speeds. In these phases, plantar flexors are highly active, providing a potential source of cross-talk. This suggests that defining onset/offset may be affected by cross-talk in these inactive periods. Additionally, at slow and preferred speed walking we also found a difference in the pre-swing phase, which may be due to the speed-effects mentioned above.

### Limitations

Sample size in intramuscular EMG studies is relatively low in general, mainly due to the invasive and costly nature of the study. Similarly, in the current study, the relatively low sample size might have led to increased type II error rate and increased uncertainty in the magnitude of the differences. This can be seen in the 95% CIs shown in the figures comparing EMG activity acquired with the two methods. Additionally, we placed intramuscular EMG electrodes in close proximity to the surface electrodes in all muscles except FHL, where intramuscular electrodes were inserted 5–10 cm proximal to the surface electrodes, and on the lateral side of the shank due to rich vascularisation close to the surface electrodes. Therefore, potential regional differences in activation might have affected the detected differences between surface and intramuscular EMG recordings. It should also be mentioned that intramuscular EMG records from a relatively smaller number of motor units compared to surface EMG, which might have been an additional source of differences detected between EMG amplitudes acquired with the two methods. However, we placed the intramuscular EMG wires as close as possible to the surface electrodes to make sure we record from the same muscle region. Inter-electrode spacing can also affect differences between the two methods. Although an inter-electrode spacing of ∼20 mm is typical in surface EMG studies (and was used in the current study for all muscles except FHL), smaller (i.e., 10 mm) inter-electrode spacing may decrease the potential for cross-talk (De Luca et al., 2012). The application of our results may be restricted to healthy and non-injured individuals with a relatively thin subcutaneous fat layer over the examined muscles.

## Conclusion

The validity of surface EMG to measure shank muscle activity is muscle- and walking speed-specific. Our results suggest that surface EMG is generally a suitable method of measuring muscle activity in MG and LG across several walking speeds. SOL and TA activity measured with surface EMG should be interpreted with caution in relevant sub-phases of walking. For FHL, surface EMG is not recommended. Future studies should explore potential sources of cross-talk and whether they can be further minimised (e.g., by decreasing inter-electrode distance), thereby improving the ability to selectively record from each shank muscle.

## Data Availability Statement

The raw data supporting the conclusions of this manuscript will be made available by the authors, without undue reservation, to any qualified researcher.

## Ethics Statement

The study was approved by the Stockholm Regional Ethic Committee (Approval No: 2017/261-31/4) and was performed in agreement with the Declaration of Helsinki.

## Author Contributions

AP, EA, TF, AH, NC, and AA conceived and designed the study, interpreted the study results, and edited and revised the manuscript. AP, EA, AH, OT, HG, and AA performed the experiments. AP and AH analysed the data and prepared the figures. AP drafted the manuscript. All authors approved the final version of the manuscript.

## Conflict of Interest

The authors declare that the research was conducted in the absence of any commercial or financial relationships that could be construed as a potential conflict of interest.

## Footnotes

1 www.spm1d.org

## Abbreviations

95% CI: 95% confidence intervals
EMG: electromyography
FHL: flexor hallucis longus
HC: heel contact
LG: lateral gastrocnemius
MG: medial gastrocnemius
SOL: soleus
SPM: Statistical Parametric Mapping
TA: tibialis anterior
TO: toe-off.

## References

[B1] Adler R. J. Taylor J. E. (2007). *Random Fields and Geometry.* 1st Edn. New York, NY: Springer-Verlag.

[B2] Andersson E. A. Nilsson J. Thorstensson A. (1997). Intramuscular emg from the hip flexor muscles during human locomotion. *Acta Physiol. Scand.* 161 361–370. 10.1046/j.1365-201X.1997.00225.x 9401589

[B3] Bogey R. Cerny K. Mohammed O. (2003). Repeatability of wire and surface electrodes in gait. *Am. J. Phys. Med. Rehabil.* 82 338–344. 10.1097/01.PHM.0000064717.90796.7A 12704271

[B4] Bogey R. A. Perry J. Bontrager E. L. Gronley J. K. (2000). Comparison of across-subject EMG profiles using surface and multiple indwelling wire electrodes during gait. *J. Electromyogr. Kinesiol.* 10 255–259. 10.1016/S1050-6411(00)00015-18 10969199

[B5] Bojsen-Moller J. Schwartz S. Kalliokoski K. K. Finni T. Magnusson S. P. (2010). Intermuscular force transmission between human plantarflexor muscles in vivo. *J. Appl. Physiol.* 109 1608–1618. 10.1152/japplphysiol.01381.2009 20884838

[B6] Chapman A. R. Vicenzino B. Blanch P. Knox J. J. Hodges P. W. (2010). Intramuscular fine-wire electromyography during cycling: repeatability, normalisation and a comparison to surface electromyography. *J. Electromyogr. Kinesiol.* 20 108–117. 10.1016/j.jelekin.2008.11.013 19339199

[B7] Cronin N. J. Avela J. Finni T. Peltonen J. (2013). Differences in contractile behaviour between the soleus and medial gastrocnemius muscles during human walking. *J. Exp. Biol.* 216(Pt 5), 909–914. 10.1242/jeb.078196 23197091

[B8] Cronin N. J. Kumpulainen S. Joutjärvi T. Finni T. Piitulainen H. (2015). Spatial variability of muscle activity during human walking: the effects of different EMG normalization approaches. *Neuroscience* 300 19–28. 10.1016/j.neuroscience.2015.05.003 25967267

[B9] Cuccurullo S. (ed.) (2004). *Physical Medicine and Rehabilitation Board Review.* New York, NY: Springer Publishing Company.

[B10] De Luca C. J. (1997). The use of surface electromyography in biomechanics. *J. Appl. Biomech.* 13 135–163. 10.1123/jab.13.2.135

[B11] De Luca C. J. Kuznetsov M. Gilmore L. D. Roy S. H. (2012). Inter-electrode spacing of surface EMG sensors: reduction of crosstalk contamination during voluntary contractions. *J. Biomech.* 45 555–561. 10.1016/j.jbiomech.2011.11.010 22169134

[B12] De Luca C. J. Merletti R. (1988). Surface myoelectric signal cross-talk among muscles of the leg. *Electroencephalogr. Clin. Neurophysiol.* 69 568–575. 10.1016/0013-4694(88)90169-90161 2453334

[B13] Dimitrova N. A. Dimitrov G. V. Nikitin O. A. (2002). Neither high-pass filtering nor mathematical differentiation of the EMG signals can considerably reduce cross-talk. *J. Electromyogr. Kinesiol.* 12 235–246. 10.1016/S1050-6411(02)00008-1 12121680

[B14] Farina D. Merletti R. Nazzaro M. Caruso I. (2001). Effect of joint angle on EMG variables in leg and thigh muscles. *IEEE Eng. Med. Biol. Mag.* 20 62–71. 10.1109/51.982277 11838260

[B15] Farina D. Negro F. (2012). Accessing the neural drive to muscle and translation to neurorehabilitation technologies. *IEEE Rev. Biomed. Eng.* 5 3–14. 10.1109/RBME.2012.2183586 23231985

[B16] Friston K. (2007). “Statistical Parametric Mapping,” in *Statistical Parametric Mapping: The Analysis of Functional Brain Images*, eds Nichols T. E. Kiebel S. J. Ashburner J. T. Penny W. D. Friston K. J., (Amsterdam: Elsevier).

[B17] Gray E. G. Basmajian J. V. (1968). Electromyography and cinematography of leg and foot (“normal” and flat) during walking. *Anat. Rec.* 161 1–15. 10.1002/ar.1091610101 5664082

[B18] Hermens H. J. Freriks B. Disselhorst-Klug C. Rau G. (2000). Development of recommendations for SEMG sensors and sensor placement procedures. *J. Electromyogr. Kinesiol.* 10 361–374. 10.1016/S1050-6411(00)00027-24 11018445

[B19] Hodges P. W. Gandevia S. C. (2000). Pitfalls of intramuscular electromyographic recordings from the human costal diaphragm. *Clin. Neurophysiol.* 111 1420–1424. 10.1016/S1388-2457(00)00341-342 10904223

[B20] Hofmann C. L. Okita N. Sharkey N. A. (2013). Experimental evidence supporting isometric functioning of the extrinsic toe flexors during gait. *Clin. Biomech.* 28 686–691. 10.1016/j.clinbiomech.2013.05.006 23735778

[B21] Kadaba M. P. Wootten M. E. Gainey J. Cochran G. V. B. (1985). Repeatability of phasic muscle activity: performance of surface and intramuscular wire electrodes in gait analysis. *J. Orthop. Res.* 3 350–359. 10.1002/jor.1100030312 4032106

[B22] Lelas J. L. Merriman G. J. Riley P. O. Kerrigan D. C. (2003). Predicting peak kinematic and kinetic parameters from gait speed. *Gait Posture* 17 106–112. 10.1016/S0966-6362(02)00060-7 12633769

[B23] Lowery M. M. Stoykov N. S. Kuiken T. A. (2003). A simulation study to examine the use of cross-correlation as an estimate of surface EMG cross talk. *J. Appl. Physiol.* 94 1324–1334. 10.1152/japplphysiol.00698.2002 12471047

[B24] Masood T. Kalliokoski K. Magnusson S. P. Bojsen-Moller J. Finni T. (2014). Effects of 12-wk eccentric calf muscle training on muscle-tendon glucose uptake and SEMG in patients with chronic achilles tendon pain. *J. Appl. Physiol.* 117 105–111. 10.1152/japplphysiol.00113.2014 24855138

[B25] Merletti R. Rainoldi A. Farina D. (2001). Surface electromyography for noninvasive characterization of muscle. *Exerc. Sport Sci. Rev.* 29 20–25. 10.1097/00003677-200101000-200101005 11210442

[B26] Neptune R. R. Kautz S. A. Zajac F. E. (2001). Contributions of the individual ankle plantar flexors to support, forward progression and swing initiation during walking. *J. Biomech.* 34 1387–1398. 10.1016/S0021-9290(01)00105-101 11672713

[B27] Neptune R. R. Sasaki K. Kautz S. A. (2008). The effect of walking speed on muscle function and mechanical energetics. *Gait Posture* 28 135–143. 10.1016/j.gaitpost.2007.11.004 18158246 PMC2409271

[B28] Onishi H. Yagi R. Akasaka K. Momose K. Ihashi K. Handa Y. (2000). Relationship between EMG signals and force in human vastus lateralis muscle using multiple bipolar wire electrodes. *J. Electromyogr. Kinesiol.* 10 59–67. 10.1016/S1050-6411(99)00020-26 10659450

[B29] Osis S. T. Hettinga B. A. Ferber R. (2016). Predicting ground contact events for a continuum of gait types: an application of targeted machine learning using principal component analysis. *Gait Posture* 46 86–90. 10.1016/j.gaitpost.2016.02.021 27131183

[B30] Õunpuu S. DeLuca P. A. Bell K. J. Davis R. B. (1997). Using surface electrodes for the evaluation of the rectus femoris, vastus medialis and vastus lateralis muscles in children with cerebral palsy. *Gait Posture* 5 211–216. 10.1016/S0966-6362(96)01087-1089

[B31] Perry J. (1992). *Gait Analysis: Normal and Pathological Function.* New Jersey, NJ: SLACK Incorporated.

[B32] Perry J. Schmidt Easterday C. Antonelli D. J. (1981). Surface versus intramuscular electrodes for electromyography of superficial and deep muscles. *Phys. Ther.* 61 7–15. 10.1093/ptj/61.1.7 7454803

[B33] Péter A. Hegyi A. Finni T. Cronin N. J. (2017). In vivo fascicle behavior of the flexor hallucis longus muscle at different walking speeds. *Scand. J. Med. Sci. Sport.* 27 1716–1723. 10.1111/sms.12810 28156022

[B34] Péter A. Hegyi A. Stenroth L. Finni T. Cronin N. J. (2015). EMG and force production of the flexor hallucis longus muscle in isometric plantarflexion and the push-off phase of walking. *J. Biomech.* 48 3413–3419. 10.1016/j.jbiomech.2015.05.033 26100463

[B35] Rainoldi A. Melchiorri G. Caruso I. (2004). A method for positioning electrodes during surface EMG recordings in lower limb muscles. *J. Neurosci. Methods* 134 37–43. 10.1016/j.jneumeth.2003.10.014 15102501

[B36] Rainoldi A. Nazzaro M. Merletti R. Farina D. Caruso I. Gaudenti S. (2000). Geometrical factors in surface EMG of the vastus medialis and lateralis muscles. *J. Electromyogr. Kinesiol.* 10 327–336. 10.1016/S1050-6411(00)00024-29 11018442

[B37] Roy S. H. De Luca C. J. Schneider J. (1986). Effects of electrode location on myoelectric conduction velocity and median frequency estimates. *J. Appl. Physiol.* 61 1510–1517. 10.1152/jappl.1986.61.4.1510 3781964

[B38] Sadoyama T. Masuda T. Miyano H. (1985). Optimal conditions for the measurement of muscle fibre conduction velocity using surface electrode arrays. *Med. Biol. Eng. Comput.* 23 339–342. 10.1007/BF02441587 4046653

[B39] Sutherland D. H. (2001). The evolution of clinical gait analysis part l: kinesiological EMG. *Gait Posture* 14 61–70. 10.1016/s0966-6362(01)00100-x 11378426

[B40] Ward S. R. Eng C. M. Smallwood L. H. Lieber R. L. (2009). Are current measurements of lower extremity muscle architecture accurate. *Clin. Orthop. Relat. Res.* 467 1074–1082. 10.1007/s11999-008-0594-598 18972175 PMC2650051

[B41] Winchester P. K. Carollo J. J. Wrobbel J. M. (1996). Reliability of gait temporal distance measures in normal subjects with and without EMG electrodes. *Gait Posture* 4 21–25. 10.1016/0966-6362(95)01032-7

